# Therapeutical strategies in cavitary legionnaires’ disease, two cases from the field and a systematic review

**DOI:** 10.1186/s12941-023-00652-5

**Published:** 2023-11-29

**Authors:** Marco Moretti, Lisanne De Boek, Bart Ilsen, Thomas Demuyser, Eef Vanderhelst

**Affiliations:** 1 Department of Internal Medicine and Infectious Diseases, Vrije Universiteit Brussel (VUB), Universitair ziekenhuis Brussel (UZB), Brussels, Belgium; 2 The ESCMID Study Group for Legionella infections (ESGLI), Gerbergasse 14, Basel, 4001 Switzerland; 3 Department of Radiology, Vrije Universiteit Brussel (VUB), Universitair ziekenhuis Brussel (UZB), Brussels, Belgium; 4 Department of Microbiology, Universitair ziekenhuis Brussel (UZB), Brussels, Belgium; 5 Faculty of Medicine and Pharmacy, AIMS Lab, Center for Neurosciences, Vrije Universiteit Brussel (VUB), Brussels, Belgium; 6 Department of Respiratory Medicine, Vrije Universiteit Brussel (VUB), Universitair ziekenhuis Brussel (UZB), Brussels, Belgium

**Keywords:** Legionnaires’ Disease, Lung abscess, Pulmonary cavitation, Combination therapy, Anaerobic coverage

## Abstract

**Background:**

Legionnaires’ Disease (LD) rarely evolves into pulmonary abscesses. The current systematic review has been designed to explore therapeutical strategies in pulmonary cavitary LD.

**Methods:**

A research strategy was developed and applied to the databases Embase, Pubmed, and Web of Science from the 1st of January 2000 to the 1st of November 2022. Original articles, case series, case reports, and guidelines written in English, French, German, Italian, and Dutch were considered. Furthermore, medical records of patients treated at the University Hospital UZ Brussel for LD cavitary pneumonia, between the 1st of January 2016 to the 1st of January 2022, were reviewed.

**Results:**

Two patients were found by the UZ Brussel’s medical records investigation. Through the literature review, 23 reports describing 29 patients, and seven guidelines were identified. The overall evidence level was low.

**Result of synthesis (case reports):**

The median age was 48 years and 65% were male. A polymicrobial infection was detected in 11 patients (44%) with other aerobic bacteria being the most commonly found. At diagnosis, 52% of patients received combination therapy, and fluoroquinolones were the preferred antimicrobial class. Anaerobic coverage was neglected in 33% of patients.

**Result of synthesis (guidelines):**

Three guidelines favor monotherapy with fluoroquinolones or macrolides, while one suggested an antimicrobial combination in case of severe LD. Four guidelines recommended anaerobic coverage in case of lung abscesses.

**Conclusion:**

To date, the evidence supporting cavitary LD treatment is low. Monotherapy lowers toxicity and might be as effective as combination therapy. Finally, anaerobes should not be neglected.

**Graphical Abstract:**

**Figure:**
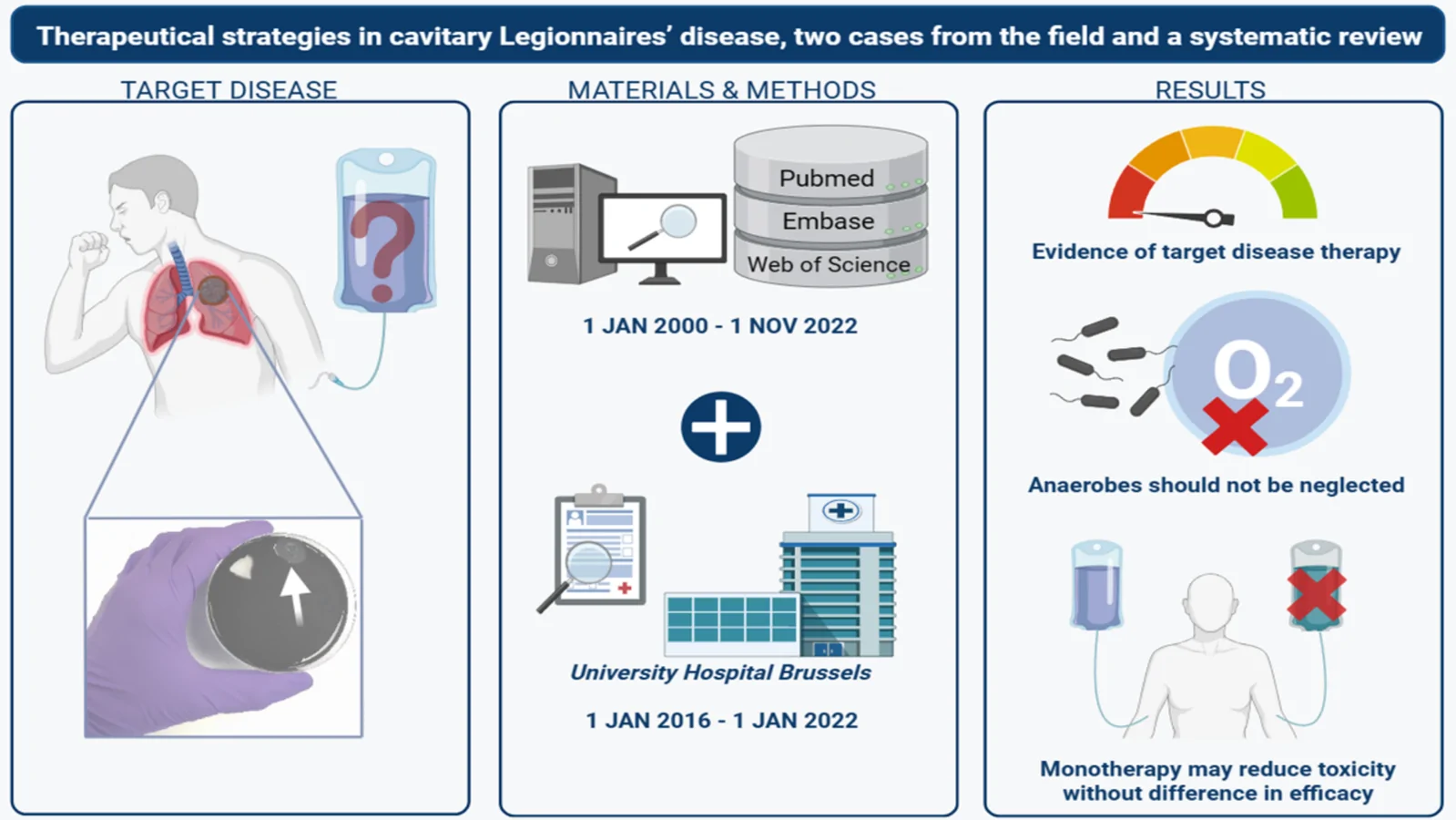

**Supplementary Information:**

The online version contains supplementary material available at https://doi.org/10.1186/s12941-023-00652-5.

## Introduction

Legionnaires’ Disease (LD) is caused by the aerobic, non-spore-forming, gram-negative bacterium *Legionella pneumophila.* Among the different *Legionella* species, *Legionella pneumophila* serogroup 1 (Lps1) is the most frequent pathogen in LD [1]. However, other species within the genus *Legionella* may provoke disease [1–3]. LD rarely evolves into pulmonary cavitary disease [4–6]. This unfavorable outcome has been associated with the depletion of cellular immunity, especially in patients with a history of hematologic malignancy or those receiving high-dose steroid treatment [6, 7]. Nevertheless, it might affect also a non-immunocompromised host [8, 9]. The preferred diagnostic method is *Legionella* urinary antigen test (LUA) with 9,566 / 10,723 (89%) cases being diagnosed by this test in Europe in 2021 [1]. However, commercially available LUA specifically detects infection due to Lps1, and their sensitivity in case of non-Lps1 infections is around 74–79% [10]. As it is estimated that 71% of LD in immunocompromised patients are caused by a non-Lps1 strain [2, 7], laboratory diagnosis of LD could be particularly challenging in these patients. Anaerobic bacteria are recognized as pathogens in lung abscesses, but their contribution to cavitary LD pathogenesis is still uncertain [11]. Moreover, there are conflicting guidelines on the preferred antibiotic regimen to treat severe LD, comprising lung abscess, with some authors recommending combination therapy and others monotherapy [10, 12–14]. Finally, it is still unclear whether the combination of some effective antimicrobials results in synergism or antagonism.

### Study objectives

This systematic review aims to analyze the treatment of cavitary pneumonia caused by *Legionella* by investigating the literature of the last 22 years. In addition, the guidelines for *Legionella* antimicrobial therapy published in the same period were revised.

## Materials and methods

### Systematic review

The present article was written using the Prisma 2020 checklist [15]. The study protocol was not registered in any public registries.

#### Eligibility criteria

All original articles, case reports, case series describing adult human cases of pulmonary cavity LD were considered. A patient affected by pulmonary cavity LD was defined as an individual 18 years of age or older who tested positive for *Legionella* using LUA, bacterial culture, or molecular testing, along with acute respiratory symptoms and a chest computed tomography (CCT) showing a lung abscess. Manuscripts describing patients who did not meet this definition were excluded, as well as reports that did not provide information on patient comorbidity, diagnostic methods, and therapeutical options. Furthermore, guidelines articles about LD management and/or lung abscess were retained. Since specific guidelines for the treatment of cavitary LD are lacking, we have extracted information on the management of severe LD and lung abscesses. Editorials, letters, comments, and non-full text articles were excluded as they provide an incomplete description of illustrated cases, focusing only on one particular aspect of the disease. Only articles in English, French, German, Italian, and Dutch were considered.

#### Search strategy and information source

The following research strategy was used: (Necrotizing Pneumonia OR Lung Abscess* OR Cavitary) AND (Legionnaire* Disease OR Infection* Legionella). This research was designed to detect the highest number of relevant articles. The research strategy was applied to the databases Embase, Pubmed (Medline), and Web of Science, covering the period from the 1st of January 2000 to the 1st of November 2022. In line with our eligibility criteria, only original articles, case reports, case series, and guidelines written in the previously defined language were considered. The research strategy of each database is detailed in the supplementary materials of this study, together with the interface or platform through which the database was searched (Supp. Materials Table 1).

#### Selection and data collection process

The systematic research of the literature was performed by one study author, who identified relevant articles through title/abstract screening (LdB). The full-text reading and data collection was completed by another study author (MM), who confirmed the correctness of the cavitary LD diagnosis in the described cases (Eligibility criteria). Manuscripts were read in their original language, and no translation to other languages occurred. No machine learning algorithm or data collection automation tool was used.

#### Data items

Following the standard of the University Hospital UZ Brussel hematologic laboratory department, leukocytopenia was defined as a leukocyte count inferior to 4300/mm^3^ and leukocytosis greater than 9600/mm^3^. Hyponatremia was delineated as sodium inferior to 136mmol/L. The articles describing patients infected by *Tatlockia micdadei*, previously known as *Legionella micdadei*, were included in this systematic review. Furthermore, the therapeutic delay was measured in days and represents the number of days between presentation at the hospital and the beginning of any effective antibiotics against *Legionella*. The therapeutic deferment was not measured from the onset of symptoms to have a more accurate number, as symptoms are subjective. Furthermore, this computation was performed to underline a potential diagnostical delay during hospitalization. Finally, no hospital-acquired LD cases were described in the current review. Only the days when an antimicrobial active against *Legionella* was administered were counted as days of treatment. The absence of anaerobic coverage was defined as the lack of any antibiotic covering anaerobic bacteria, regardless of the duration of antimicrobial treatment.

### Case series

Medical records of patients treated at UZ Brussel for cavitary pneumonia due to LD, between the 1st of January 2016 to the 1st of January 2022, were reviewed. Patients were retrieved by one study author (MM) through positive microbiology specimens for *Legionella* such as nucleic acid amplification test (NAAT), culture of respiratory specimens, or LUA. The above-reported definition of cavitary LD (Eligibility criteria) was used to confirm cases.

### Statistical analyses

Data are expressed as median and interquartile range for continuous variables, numbers, and proportions for categorical variables. No distinct groups were compared. Descriptive analyses were performed with IBM SPSS Statistics for Windows, Version 20.0. Armonk, NY: IBM Corp, released in 2011.

## Results

### UZ Brussel case series

#### First case

**Fig. 1:**
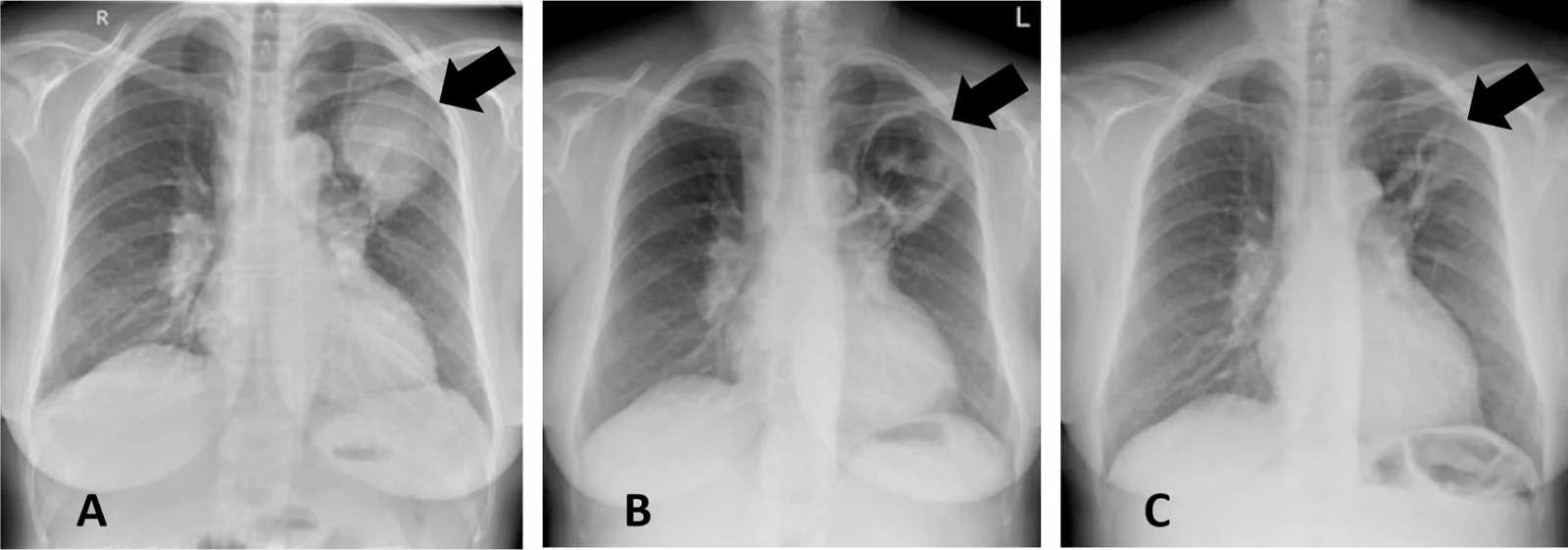
Evolution of chest imaging in a patient affected by Legionnaires’ Disease lung abscess. Evolution over time of chest X-ray of the second reported case, from right to left respectively (subpanel **A**, **B**, **C**). The lung lesion is underlined with a black arrow. **A** (left): left apical consolidation; **B** (middle): evolution of the consolidation into cavitation; **C** (right): partially resolution with shrinking of the lesion

A 74-year-old female with a medical history of multiple myeloma, which relapsed after autologous stem-cell transplantation in 2016, was treated with daratumumab, lenalidomide, and dexamethasone. The chemotherapy was administered three weeks before hospital admission. The patient presented with fever (38.7 °C tympanic) and fatigue. Blood analysis at hospitalization showed neutropenia (absolute count neutrophils was 497/mm3) and CRP of 342 mg/L. A CCT scan found an opacification of the left lower lobe with the presence of an abscess (44 × 21 mm), Supp. Materials Fig. 1. The patient was started on broad empiric antibiotics (cefepime 2 g TID and metronidazole 500 mg TID), and a bronchoscopy was ordered. Bronchoalveolar fluid (BAL) cultures were negative, but *Legionella pneumophila* serogroup 1 NAAT and the galactomannan on the same sample (index:1.12) were both positive. Furthermore, LUA was also positive. Antibiotic therapy was narrowed to moxifloxacin (MFX) 400 mg QD. As the diagnosis of probable invasive pulmonary mold diseases was met following the criteria of EORTC/MSG [16], the patient was also treated with voriconazole. A compatible CCT imaging is part of the EORTC/MSG diagnostic criteria of an invasive fungal infection. Pulmonary aspergillosis may present in an initial phase with typical signs as nodular opacities of a halo sign. However, CCT images may not differ between mold or bacterial infections in a later phase as lung cavitations appear [17]. Considering the neutropenia, Filgrastim 0.3 mg injection was given daily until complete recovery of the neutrophils absolute count. After 14 days of treatment, the patients persisted to be febrile (38.1 °C tympanic), CRP decreased to 110 mg/L and neutrophils were in the normal range (absolute count of 5003/mm3). CCT was repeated and an increase in pleura effusion and the dimension of the abscess was observed (60 × 34 mm), supplementary materials Fig. 1. A multidisciplinary discussion was organized to choose the most appropriate treatment option, involving a pneumologist, infectious disease specialist, haematologist, radiologist, and thorax surgeon. The abscess was not ripe enough to be drained, so a conservative approach was considered. A chest tube was placed to evacuate pleural effusion. Furthermore, moxifloxacin was continued for a total of 21 days, and voriconazole for six weeks. After stopping antibiotic treatment and removing the chest tube, the CCT revealed a persistent abscess and consolidation without pleural effusion. Finally, CCT was repeated four months after the diagnosis of the infection, and a complete cure of the abscess was observed (Supp. Materials Fig. 1). Chemotherapy was restarted only after these reassuring findings.

#### Second case

A 55-year-old female with a medical history of asthma on inhaled steroids and type 2 diabetes on insulin therapy was diagnosed with systemic sarcoidosis three weeks before admission. The disease involved mediastinal, peri-hepatic, and splenic lymph nodes. Treatment with high-dose methylprednisolone (64 mg QD) was initiated. She presented with a dry cough and haemoptysis. Blood analysis found a CRP of 231 mg/L and leucocytosis (14,500/mm3); chest X-ray showed opacification left upper lobe (Fig. 1). Bronchoscopy was performed, and the BAL sample was analyzed. The sample tested positive for *Legionella pneumophila* serogroup 1 NAAT. Therefore, MFX 400 mg QD was started, and methylprednisolone was reduced to 32 mg QD. Five days after admission, the patient was afebrile and did not require oxygen. She was discharged with a close follow-up, 10 days after discharge. At that time, she reported persistent cough and chest pain, and inflammatory markers were decreased (CRP of 12 mg/L and white blood cell count of 11,400/mm3). A chest X-ray was obtained, which suggested an evolution into cavitary pneumonia (Fig. 1). This suspicion was confirmed by CCT. Therefore, methylprednisolone was decreased to 16 mg QD, and antibiotic treatment was continued for 21 days in total with favourable clinical and radiological evolution (Fig. 1).

### Systematic review

#### Study selection and characteristics

**Fig. 2:**
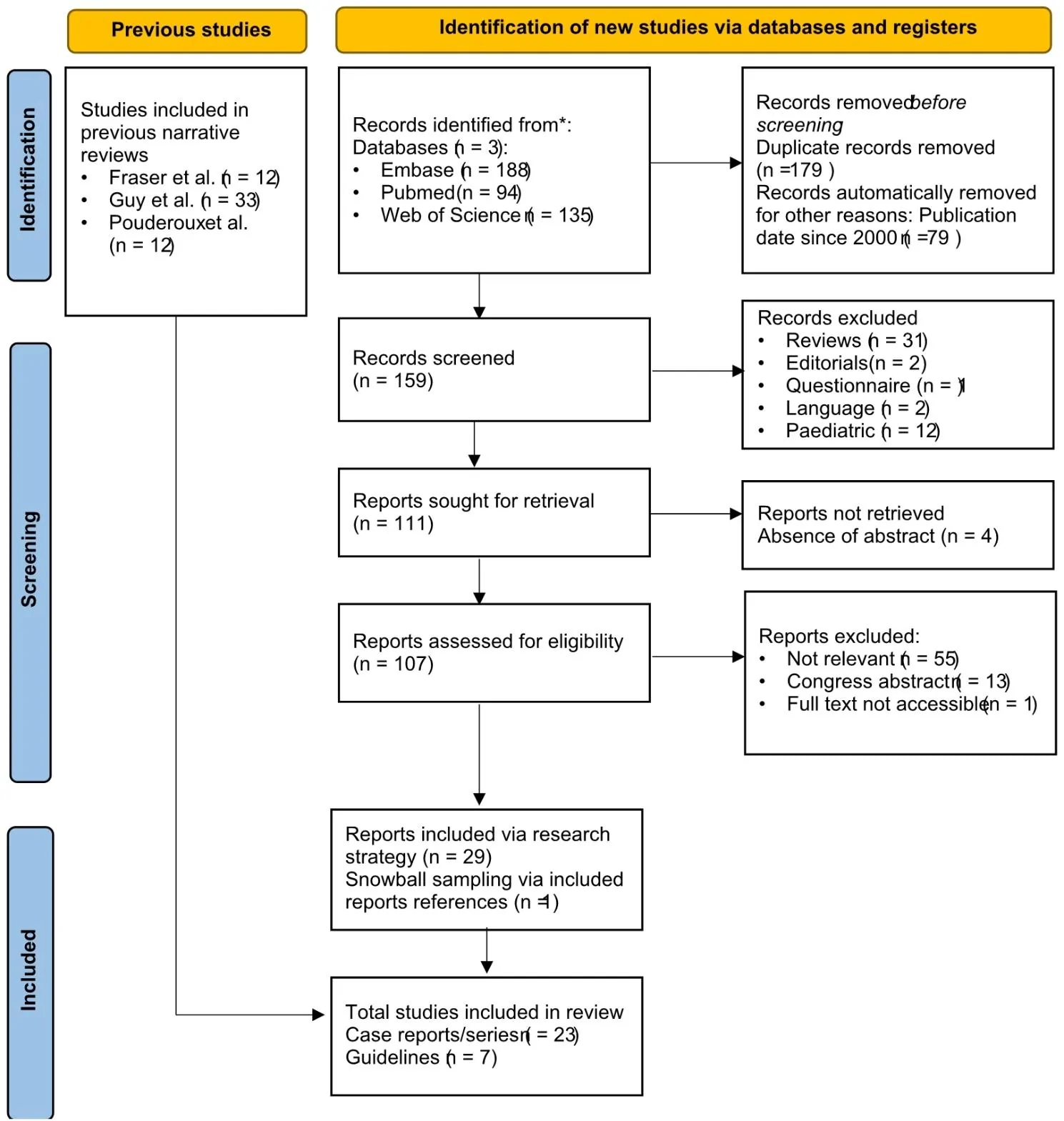
Study flowchart. Edition PRISMA 2020 flow diagram

The PRISMA 2020 flow diagram is shown in Fig. 2. Following the screening procedure, 23 case series or case reports, and six guidelines were included. After reading the full text of the guidelines, one additional guideline was found through the references of the guidelines selected by the research strategy [18]. Table 1 lists the included guidelines. Supp. Materials Table 2 depicts the selected manuscripts and the number of the described patients per article.

**Table 1:** Considered guidelines and their recommendations

First author, journal and year	Title	Recomandations
Höffken et al., Pneumologie 2005	S3-guideline on ambulant acquired pneumonia and deep airway infections	***LD therapy***: IV ERY or oral fluoroquinolones (e.g. LVX 750 mg QD). MFX and LVX are equal and superior to CLA in cellular culture infection models. ***Lung abscess treatment***: Bronchoscopy should be performed (diagnostic and therapeutic). IV therapy should be chosen. Peroral switch when clinical or radiological improvements are noticed. Anaerobes are involved in 20–90% of cases and should be covered. Therapy should be continued until radiographic resolution.
Lim et al., Thorax 2009 (2015 - Annotated)	2015 - Annotated BTS Guideline for the management of CAP in adults (2009) Summary of recommendations	***LD therapy***: For low and moderate severity, an oral fluoroquinolone is recommended. In the unusual case when this is not possible due to patient intolerance, a macrolide is an alternative. For the management of high severity, a fluoroquinolone is recommended. For the first few days this can be combined with a macrolide (AZM is an option in countries where it is used for pneumonia) or rifampicin as an alternative. Clinicians should be alert to the potential small risk of cardiac electrophysiological abnormalities with quinolone-macrolide combinations.
Bartolf et al., Medicine 2016	Pneumonia	Not covered, reference to Lim et al.
Mikasa et al., J Infect Chemother 2016	JAID/JSC Guidelines for the Treatment of Respiratory Infectious Diseases: The Japanese Association for Infectious Diseases/Japanese Society of Chemotherapy - The JAID/JSC Guide to Clinical Management of Infectious Disease/Guideline-preparing Committee Respiratory Infectious Disease WG	***LD therapy***: In case of inpatients, the first choices: IV LVX 500 mg QD, IV CIP 300 mg BID of TID, IV PZFX 500-1,000 mg BID, IV AZM 500 mg QD. The second choice is IV ERY 500 mg TID plus RIF (oral) 450-600 mg QD. Outpatient treatment is oral with the same drugs. ***Lung abscess treatment***: In lung abscess, anaerobes are primarily involved.
Athlin et al., Infect Dis (Lond) 2018	Management of community-acquired pneumonia in immunocompetent adults: updated Swedish guidelines 2017	***LD therapy***: Recommended regimens are: LVX 750 mg q.d. or MFX 400 mg q.d. or AZM 500 mg q.d. ***Lung abscess treatment***: anaerobic coverage is reccommended (e.g. b-lactam/b-lactamase inhibitor or MFX). Treatment duration should be at least 6–8 weeks.
Metlay et al., Am J Respir Crit Care Med 2019	Diagnosis and Treatment of Adults with Community-acquired Pneumonia. An Official Clinical Practice Guideline of the American Thoracic Society and Infectious Diseases Society of America	***Lung abscess treatment***: Anaerobic coverage is suggested for patients diagnosed with lung abscess or empyema
Gauzit et al., Infect Dis Now 2021	Anti-infectious treatment duration: The SPILF and GPIP French guidelines and recommendations	Not covered, reference to Lim et al. and Metlay et al.

AZM: azithromycin; CAP: community acquired pneumonia; CIP: ciprofloxacin; CLA: clarithromycin; ERY: erythromycin; LD: Legionnaires’ disease; IV: intravenous; LUA: *Legionella* urinary antigen test; LVX: levofloxacin; MFX: moxifloxacin; NAAT: nucleic acid amplification test

#### Result of synthesis (case reports)

Table 2 illustrates the descriptive characteristics of the 29 patients found by the systematic review. The median age was 48 years and 65% were male. The most reported comorbidity was hematologic malignancy (n:8 61%), and the most prevalent immunosuppressant treatment was corticosteroids (n:17 85%). The preferred diagnostic method was bacterial culture, mostly performed on BAL fluid or lung tissue biopsies, with a sensitivity of 86% (4 false negatives). This was followed by LUA testing, which was performed for 25 patients and had a sensitivity of 52% (12 false negatives, only patients with LD with non-Lps1 pathogenic agent). Lps1 was the most commonly isolated species of *Legionella*, affecting 52% of the described patients. A delay of the beginning of an effective therapy superior to five days or more occurred in 56% of the patients. After diagnosis, bi-therapy with two or more effective antimicrobials was started in 52% of the patients. Supp. Materials Table 3 illustrates the antimicrobial regimen adaption after diagnosis of LD abscess. Fluoroquinolones were the preferred antibiotic class, with 76% of the patients being treated with one antimicrobial from this class. A co-infection diagnosed through isolation of a co-pathogen occurred in 11 cases, with four patients growing an anaerobic bacterium. Particularly, two times both*Fusobacterium nucleatum* and *Prevotella* species [7, 9, 19, 20]. In case of polymicrobial flora, isolates were cultured in lung biopsy (two patients), protected bronchial brush specimen (one patient), empyema sample (two patients), BAL fluid (five patients), and finally one patient had a sputum sample growing *Enterococcus faecalis* and a positive LUA. An absence of anaerobic coverage was observed in 33% of the patients. The median duration of therapy was 42 days. Frequently the consolidation evolved into empyema, and 59% of the patients needed chest tube drainage. Thoracic surgery was performed in 22% of the patients: two lobectomies, one wedge resection, and two abscess resections. One operation was not in detail described. The reported reasons for surgery were clinical deteriorations (increased pain and inflammation markers, increase in abscess volume, and appearance of empyema) and in two cases unclear diagnostic prompted surgical management [9, 21]. All-cause mortality was 21%.

**Table 2:** Characteristics of cavity Legionnaires’ disease patients found by systematic review

Legionnaires’ Disease patients (n: 29)	
Demographic	Gender, male: 19 (65%) Age, years: 48 (34–63) Active smoking: 3 (33%)
Comorbidities: 11 (38%)	Connective tissue diseases: 4 (36%) Solid organ transplantation: 2 (18%) Inflammatory bowel diseases: 2 (18%) Diabetes: 1 (9%) Asthma: 1 (9%) COPD: 0 (0%)
Malignancy: 13 (45%)	Hematologic malignancies: 8 (61%) Solid cancers: 5 (38%)
Immunosuppressive treatment: 20 (69%)	Steroid: 17 (85%) Chemotherapy: 9 (45%) Single immunomodulators*: 8 (40%)
Blood tests	Leukocytosis: 11 (52%) Leukocytopenia: 5 (24%) Hyponatremia: 6 (30%)
Diagnostic	Positive respiratory sample culture: 25 (86%) Positive *Legionella* urinary antigen test: 13 (52%) Positive molecular tests: 8 (15%)
*Legionella* species	Serotype 1 *Legionella pneumophila*: 15 (52%) Non-Serotype 1 *Legionella pneumophila*: 3 (10%)
Co-infection: 11 (44%)	Aerobic bacteria 5 (45%) Anaerobic bacteria 4 (36%) Aspergillus: 2 (18%)
Antimicrobial regimens	Upfront combination therapy after diagnosis: 15 (52%) Length of treatment: 42 (21–59) Absence of any anaerobic coverage: 8 (33%)
Antimicrobial regimens	Treatment comprising a fluoroquinolone: 22 (76%) Treatment comprising a macrolide: 21 (72%) Treatment comprising rifampicin: 9 (31%)
Delay in effective antibacterial treatment	> 5 days: 15 (56%) > 10 days: 10 (37%) Post-mortem diagnosis: 1 (3%)
Additional therapy	Chest tube drainage: 16 (59%) Thoracic surgery: 6 (22%)
Disease severity	Intensive care unit admission: 10 (43%) All-cause mortality: 6 (21%)

*Patients were treated with only one immunomodulatory drug with or without steroids association, (e.g. methotrexate + / - steroids)

#### Result of synthesis (guidelines)

Four guidelines provided suggestions for LD treatment [12–14, 20]. Three reports proposed monotherapy [12, 13, 22], while one suggested combination treatment [14]. Furthermore, fluoroquinolones and macrolides were mentioned as the first antibiotic choice in four guidelines [12–14, 20]. Finally, two guidelines advocated for initial intravenous therapy before possible oral relay [13, 22].

Four guidelines discussed therapy for lung abscesses, and all four reports suggested the potential pathogenic role of anaerobes [12, 13, 18, 22]. One article proposed a length of treatment of six to eight weeks [12], while another suggested treatment until radiological resolution [22].

#### Certainty of the evidence reported in the guidelines

Recommendations for LD antimicrobial therapy are based on observational reports [21, 23–25]. In a prospective study conducted in Spain during an outbreak, there was no difference in clinical outcome or length of hospitalisation between patients treated with fluoroquinolones and macrolides (292 patients). However, in severe patients, treatment with levofloxacin (LVX) reduced complications and length of stay [23]. Another prospective study observed 45 severe LD patients and found no improvement when using a combination of LVX and rifampicin (RIF) compared to LVX monotherapy [24]. Finally, a study on 25 patients found increased mortality in those treated with monotherapy compared to the combination of clarithromycin and RIF [25].

Anaerobes are one of the main pathogens involved in lung abscesses. A study in Taiwan found anaerobes in 34% of the specimens obtained from 90 patients [11]. Guidelines for managing lung abscesses rely on RCTs [24, 26–28]. RCTs comparing the management of pulmonary abscess with antimicrobials without anaerobic coverage resulted in lower clinical cure rates than therapy with an anaerobic spectrum [26, 27]. Additionally, an RCT comparing MFX and ampicillin-sulbactam in 139 patients for the treatment of aspiration pneumonia and pulmonary abscess reported similar clinical outcomes. The median duration of MFX therapy for lung cavitation was 30 days, and the adverse events were comparable between the two treatment arms [28].

## Discussion

The current manuscript presents two cases of pulmonary cavitary LD treated at the UZ Brussel. Furthermore, it describes the clinical features of 29 cases retrieved by a literature review together with the recommendations of seven guidelines, and the scientific evidence on which they are based.

In the current report, the majority of affected patients were male. However, none of them had a medical history of COPD, and only three patients were actively smoking [1, 2]. Affected patients, frequently had a medical history of malignancy, particularly hematologic, and immunosuppressive therapy as steroids or chemotherapy. Further prospective larger studies should assess if typical risk factors of LD, such as tobacco smoking or COPD, are less prominent in patients diagnosed with lung cavitations due to *Legionella*.

LUA is the main diagnostic method and is frequently considered the diagnostic golden standard in severe pneumonia patients by guidelines [29, 30]. However, it might have insufficient sensitivity in case of pulmonary cavitary LD. Nevertheless, it should be systematically performed as Lps1 is the most prevalent pathogen in this form of disease [6, 7]. Bronchoscopy with BAL fluid examination is frequently part of the work-up to rule out opportunistic molds (e.g. *Aspergillus* spp.) and slowly growing bacteria (e.g. *Mycobacterium* spp. and *Nocardia* spp.). *Legionella* NAAT on BAL fluid may increase sensitivity and might reduce the time to diagnosis and avoid therapeutic delay. In two of the six LD cases requiring thoracic surgery, diagnostic uncertainty together with clinical deterioration prompted surgery [9, 21]. Prompt diagnosis through NAAT on BAL fluid might have triggered a rapid beginning of effective therapy and spared the patients an invasive procedure. However, only retrospective studies indicate a possible sensitivity advantage of molecular testing, and further evidence is needed before routine use [31, 32].

*Legionella* is a fastidious bacterium that is not considered to be a human commensal. Its presence may suggest disease [33, 34]. The analysis of the sputum microbiome in patients affected by pneumonia showed a polymicrobial flora. In some cases, *Legionella* was found to be only a minority of the total amount of bacteria in patients diagnosed with LD [35, 36]. This bacterium is more difficult to grow than other co-pathogens. However, it is possible that *Legionella* might require specific conditions generated by other co-pathogens in order to cause disease. In the described case series, 44% of the isolates were part of a polymicrobial flora with almost all the microbes (91%) being grown in deep respiratory samples. To date, the role of each independent pathogen in the development of lung abscesses is unknown. In our opinion, attempts should be made to isolate responsible microbes in lung abscesses, considering *Legionella* testing in each affected patient undergoing deep respiratory sampling. Observational studies suggest the use of fluoroquinolones in patients with severe pneumonia [23, 24]. However, RCTs were not performed, probably due to the relative rarity of the disease. In the above-mentioned cases, fluoroquinolones were the preferred antimicrobial. Almost half of the cases were treated with combination therapy, but evidence on these regimes is limited and results are contrasting [11, 23–25]. Adverse events from drug-drug interactions and liver toxicity caused by RIF might be serious [37, 38]. Fluoroquinolones with macrolides combination have been poorly studied and potential cardiotoxicity might occur [39, 40]. Furthermore, the presented case series showed a complete overlooking of anaerobic coverage in 31% of the patients. An RCT on lung abscess compared MFX for a median of 30 days to ampicillin-sulbactam with similar outcomes [28]. This is the only RCT in lung abscess which have studied an antimicrobial that is effective on *Legionella*. We believe that monotherapy moxifloxacin might be an adequate therapy for cavitary LD, as it is an effective bactericidal agent with acceptable anaerobic coverage. Nevertheless, this antibiotic is less studied in patients affected by *Legionella* infection than other quinolones (e.g. levofloxacin) or macrolides (e.g. azithromycin), and further prospective studies should confirm the efficacy of this strategy.

### Study strengths and limitations

The strengths of this systematic review include the consideration of articles published on three databases and written in five languages, which increases the number of eligible articles and the exhaustiveness of this review. To the best of our knowledge, this is the first systematic review addressing this topic. The current study increases the evidence-based approach to lung abscesses in LD.

However, some limitations should be mentioned. The literature search, the article screening, and the data collection were performed by only one study investigator. The review of medical records at the UZ Brussel focussed specifically on the period between 2016 and 2022. In contrast, the literature review encompassed the years 2000 and 2022. The reason for this discrepancy is due to the introduction of routine NAAT on BAL fluid specimens for LD diagnosis at the UZ Brussel. Some cases of LD, particularly non-Ls1, may have been underdiagnosed in the years before 2016. Moreover, we consider this period as representative.

Some recommendations extrapolated by the guidelines were broad and were not studied in the specific context of *Legionella* infection. For example, an RCT compared MFX monotherapy with ampicillin-sulbactam for the treatment of lung abscesses. Within this RCT, no patients were diagnosed with LD, so the efficacy of MFX could only be assumed. Finally, the data of considered reports might be incomplete. For instance, some authors might not have described the antibiotic with anaerobic coverage in the case reports, as the main focus was LD.

## Conclusion

To date, evidence of cavitary LD treatment is poor. Monotherapy lowers toxicity and might be as effective as combination therapy. Finally, anaerobes should not be neglected.

### Future and prospective

Since lung abscesses in LD are infrequent, conducting RCTs may not be feasible. Instead, large prospective registers could help increase the evidence-based approach to cavitary pulmonary LD patients.

## Electronic supplementary material

Below is the link to the electronic supplementary material.

Supplementary Material 1

## Data Availability

The datasets used and/or analyzed during the current study are available from the corresponding author upon reasonable request.

## References

[1] European Centre for Disease Prevention and Control. Legionnaires’ disease - Annual Epidemiological Report for 2021. https://www.ecdc.europa.eu/en/publications-data/legionnaires-disease-annual-epidemiological-report-2021. Date of access: the 10th August 2023.

[2] Cunha BA, Burillo A. Bouza. Legionnaires’ Disease. Lancet. 2016;387(10016):376–85. 10.1016/S0140-6736(15)60078-2. 26231463

[3] Moretti M, Allard SD, Dauby N, De Geyter D, Mahadeb B, Miendje VY, et al. Clinical features of Legionnaires’ disease at three Belgian university hospitals, a retrospective study. Acta Clin Belg. 2021;1–7. 10.1080/17843286.2021.1978211. 34520336

[4] Durrance RJ, Min AK, Fabbri M, McGarry T. Cavitary Legionella Pneumonia in AIDS: when Intracellular immunity failure leads to Rapid Intrapulmonary Cavitation. Case Rep Pulmonol 2021 Nov 30:20216754094. 10.1155/2021/6754094. PMC8651395 34888109

[5] Gavand P-E, Janssen R, Martin A, Ledoux M-P, Schneider F. Persistant Legionella pneumophila and Enterococcus faecium pulmonary Infection: look for an abscess! Presse Med. 2016;45(4 Pt 1):477–9. 10.1016/j.lpm.2016.01.009. 26874909

[6] Guy SD, Worth LJ, Thursky KA, Francis PA, M. A. Slavin. Legionella pneumophila lung abscess associated with immune suppression. 2011;41(10):715 – 21. 10.1111/j.1445-5994.2011.02508.x. 22435900

[7] C. Pouderoux, C. Ginevra, G. Descours, A.-G. Ranc, L. Beraud, S. Boisset. Slowly or Nonresolving Legionnaires’ Disease: Case Series and Literature Review. *Clin Infect Dis*. 2020;70(9):1933–1940. 10.1093/cid/ciz538. 31242293

[8] Bell H, Chintalapati S, Patel P, Halim A, Kithas A. Legionella longbeachae Pneumonia: case report and review of reported cases in non-endemic countries. IDCases. 2021;23:e01050. 10.1016/j.idcr.2021.e01050. PMC7817369 33511033

[9] Descours G, Tellini C, Flamens C, Philit F, Celard M, Etienne J, et al. Legionellosis and lung abscesses: contribution of legionella quantitative real-time PCR to an adapted followup. Case Rep Infect Dis. 2013;2013:190183. 10.1155/2013/190183. PMC3686064 23862082

[10] Viasus D, Gaia V, Manzur-Barbur C, Carratalà J. Legionnaires’ Disease: Update on Diagnosis and Treatment. *Infect Dis Ther* 2022;11(3):973–986. 10.1007/s40121-022-00635-7. PMC9124264 35505000

[11] J.-L. Wang, K.-Y. Chen, C.-T. Fang, P.-R. Hsueh, P.-C. Yang, S.-C. Chang. Changing bacteriology of adult community-acquired lung abscess in Taiwan: Klebsiella pneumoniae versus anaerobes. *Clin Infect Dis*. 2005;40(7):915 – 22. 10.1086/428574. 15824979

[12] Athlin S, Lidman C, Lundqvist A, Naucler P, Nilsson AC, Spindler C, et al. Management of community-acquired Pneumonia in immunocompetent adults: updated Swedish guidelines 2017. Infect Dis (Lond). 2018;50(4):247–72. 10.1080/23744235.2017.1399316. 29119848

[13] Mikasa K, Aoki N, Aoki Y, Abe S, Iwata S, Ouchi K, et al. The JAID/JSC Guide to Clinical Management of Infectious Disease/Guideline-preparing Committee Respiratory Infectious Disease WG. J Infect Chemother. 2016;22(7 Suppl):1–S65. 10.1016/j.jiac.2015.12.019. JAID/JSC Guidelines for the Treatment of Respiratory Infectious Diseases: The Japanese Association for Infectious Diseases/Japanese Society of Chemotherapy -. PMC7128733 27317161

[14] Lim WS, Baudouin SV, George RC, Hill AT, Jamieson C, Le Jeune I, et al. 2015 - annotated BTS Guideline for the management of CAP in adults (2009) Summary of recommendations. Thorax. 2009;64:iii1–iii55. 10.1136/thx.2009.121434. 19783532

[15] Page MJ, McKenzie JE, Bossuyt PM, Boutron I, Hoffmann TC, Mulrow CD, et al. The PRISMA 2020 statement: an updated guideline for reporting systematic reviews. BMJ. 2021;372:n71. 10.1136/bmj.n71. PMC8005924 33782057

[16] Donnelly JP, Chen SC, Kauffman CA, Steinbach WJ, Baddley JW, Verweij PE, the Mycoses Study Group Education and Research Consortium. Revision and update of the Consensus definitions of Invasive Fungal Disease from the European Organization for Research and Treatment of Cancer and. Clin Infect Dis. 2020;71(6):1367–76. 10.1093/cid/ciz1008. PMC7486838 31802125

[17] Alexander BD, Lamoth F, Heussel CP, Prokop CS, Desai SR, Morrissey CO, et al. Guidance on Imaging for Invasive Pulmonary aspergillosis and mucormycosis: from the Imaging Working Group for the revision and update of the Consensus definitions of Fungal Disease from the EORTC/MSGERC. Clin Infect Dis. 2021;72(Suppl 2):79–S88. 10.1093/cid/ciaa1855. 33709131

[18] Metlay JP, Waterer GW, Long AC, Anzueto A, Brozek J, Crothers K, et al. Diagnosis and treatment of adults with community-acquired Pneumonia. An Official Clinical Practice Guideline of the American Thoracic Society and Infectious Diseases Society of America. Am J Respir Crit Care Med. 2019;200(7):e45–e67. 10.1164/rccm.201908-1581ST. PMC6812437 31573350

[19] Schindel C, Siepmann U, Han S-R, Ullmann AJ, Mayer E, Fischer T, et al. Persistent Legionella Infection in a patient after bone marrow transplantation. J Clin Microbiol. 2000;38(11):4294–5. 10.1128/jcm.38.11.4294-4295.2000. PMC87590 11060117

[20] Miyara T, Tokashiki K, Shimoji T, Tamaki K, Koide M, Saito A. Rapidly expanding lung abscess caused by Legionella pneumophila in immunocompromised patients: a report of two cases. Intern Med. 2002;41(2):133–7. 10.2169/internalmedicine.41.133. 11868601

[21] Fraser TG, Zembower TR, Lynch P, Fryer J, Salvalaggio PRO, Yeldandi AV, et al. Cavitary Legionella Pneumonia in a liver transplant recipient. Transpl Infect Dis. 2004;6(2):77–80. 10.1111/j.1399-3062.2004.00053.x. 15522109

[22] Höffken G, Lorenz J, Kern W, Welte T, Bauer T, Dalhoff K, et al. S3-guideline on ambulant acquired Pneumonia and deep airway Infections. Pneumologie. 2005;59(9):612–64. 10.1055/s-2005-870988. 16170727

[23] Blázquez Garrido RM, Parra FJE, Alemany Francés L, Guevara RMR, Sánchez-Nieto JM, Hernández MS, et al. Antimicrobial chemotherapy for Legionnaires Disease: levofloxacin versus macrolides. Clin Infect Dis. 2005;40(6):800–6. 10.1086/428049. 15736011

[24] Griffin AT, Peyrani P, Wiemken T, Arnold F. Macrolides versus quinolones in Legionella Pneumonia: results from the community-acquired Pneumonia Organization international study. Int J Tuberc Lung Dis. 2010;14(4):495–9. 20202309

[25] Rello J, Gattarello S, Souto J, Sole-Violan J, Valles J, Peredo R, et al. Community-acquired Legionella Pneumonia in the intensive care unit: impact on survival of combined antibiotic therapy. Med Intensiva. 2013 Jun-Jul;37(5):320–6. 10.1016/j.medin.2012.05.010. 22854618

[26] Levison ME, Mangura CT, Lorber B, Abrutyn E, Pesanti EL, Levy RS, et al. Clindamycin compared with penicillin for the treatment of anaerobic lung abscess. Ann Intern Med. 1983;98(4):466–71. 10.7326/0003-4819-98-4-466. 6838068

[27] Allewelt M, Schüler P, Bölcskei PL, Mauch H, Lode H. Ampicillin + sulbactam vs clindamycin +/- cephalosporin for the treatment of aspiration Pneumonia and primary lung abscess. Clin Microbiol Infect. 2004;10(2):163–70. 10.1111/j.1469-0691.2004.00774.x. 14759242

[28] Ott SR, Allewelt M, Lorenz J, Reimnitz P. Lode. Moxifloxacin vs ampicillin/sulbactam in aspiration Pneumonia and primary lung abscess. Infection. 2008;36(1):23–30. 10.1007/s15010-007-7043-6. 18231720

[29] Yzerman EPF, den Boer JW, Lettinga KD, Schellekens J, Dankert J, Peeters M. Sensitivity of three urinary antigen tests associated with clinical severity in a large outbreak of Legionnaires’ Disease in the Netherlands. J Clin Microbiol. 2002;40(9):3232–6. 10.1128/JCM.40.9.3232-3236.2002. PMC130686 12202558

[30] van der Eerden MM, Vlaspolder F, de Graaff CS, Groot T, Bronsveld W, Jansen HM et al. Comparison between pathogen directed antibiotic treatment and empirical broad spectrum antibiotic treatment in patients with community acquired pneumonia: a prospective randomised study. *Thorax*. 2005;60(8):672-8. 10.1136/thx.2004.030411. PMC1747487 16061709

[31] D. R. Murdoch. Nucleic acid amplification tests for the diagnosis of pneumonia. *Clin Infect Dis*. 2003;36(9):1162-70. 10.1086/374559. 12715312

[32] Mentasti M, Fry NK, Afshar B, Palepou-Foxley C, Naik FC, Harrison TG. Application of Legionella pneumophila-specific quantitative real-time PCR combined with direct amplification and sequence-based typing in the diagnosis and epidemiological investigation of Legionnaires’ Disease. Eur J Clin Microbiol Infect Dis. 2012;31(8):2017–28. 10.1007/s10096-011-1535-0. 22278293

[33] Muder RR, Yu VL. Fang. Community-acquired Legionnaires’ Disease. Semin Respir Infect. 1989;4(1):32–9. 2652233

[34] D. R. Murdoch. Diagnosis of Legionella infection. *Clin Infect Dis*. 2003;36(1):64 – 9. doi: 10.1086/345529. Epub 2002 Dec 12. 10.1086/345529 12491204

[35] Mizrahi H, Peretz A, Lesnik R, Aizenberg-Gershtein Y, Rodríguez-Martínez S, Sharaby Y, et al. Comparison of sputum microbiome of legionellosis-associated patients and other Pneumonia patients: indications for polybacterial Infections. Sci Rep. 2017 Jan;6:7:40114. 10.1038/srep40114. PMC5216348 28059171

[36] Pérez-Cobas AE, Ginevra C, Rusniok C, Jarraud S, Buchrieser C. The respiratory tract microbiome, the pathogen load, and clinical interventions define severity of bacterial Pneumonia. Cell Rep Med. 2023;4(9):101167. 10.1016/j.xcrm.2023.101167. PMC10518590 37633274

[37] Baciewicz AM, Chrisman CR, Finch CK, Self TH. Update on rifampin, rifabutin, and rifapentine drug interactions. Curr Med Res Opin. 2013;29(1):1–12. 10.1185/03007995.2012.747952. 23136913

[38] Grau S, Mateu-de Antonio J, Ribes E, Salvadó M, Garcés JM, Garau J. Impact of rifampicin addition to clarithromycin in Legionella pneumophila Pneumonia. Int J Antimicrob Agents. 2006;28(3):249–52. 10.1016/j.ijantimicag.2006.03.029. 16870401

[39] Polk RE. Drug-drug interactions with ciprofloxacin and other fluoroquinolones. Am J Med. 1989;87(5A):76S–81S. 10.1016/0002-9343(89)90028-4. 2686430

[40] Bastida C, Soy D, Torres A. The safety of antimicrobials for the treatment of community-acquired Pneumonia. Expert Opin Drug Saf. 2020;19(5):577–87. 10.1080/14740338.2020.1750594. 32239981

