# Supplementary material for: Therapeutical strategies in cavitary legionnaires’ disease, two cases from the field and a systematic review
Source: Ann Clin Microbiol Antimicrob. 2023 Nov 29;22:105. doi: 10.1186/s12941-023-00652-5 (PMC10687996; doi:10.1186/s12941-023-00652-5)
Supplement: Supplementary file 1 — Supplementary Material 1 [file 12941_2023_652_MOESM1_ESM.docx]

**Therapeutical strategies in cavitary Legionnaire’s disease, a systematic review**

**Supplementary materials**

***Figure legend***

***Supp. Materials Figure 1 – Evolution of chest computed tomography in a patient affected by Legionnaires’ Disease lung abscess.*** Evolution over time of chest computed tomography of the first reported case, from right to left respectively.

***Supp. Materials Figure 1***


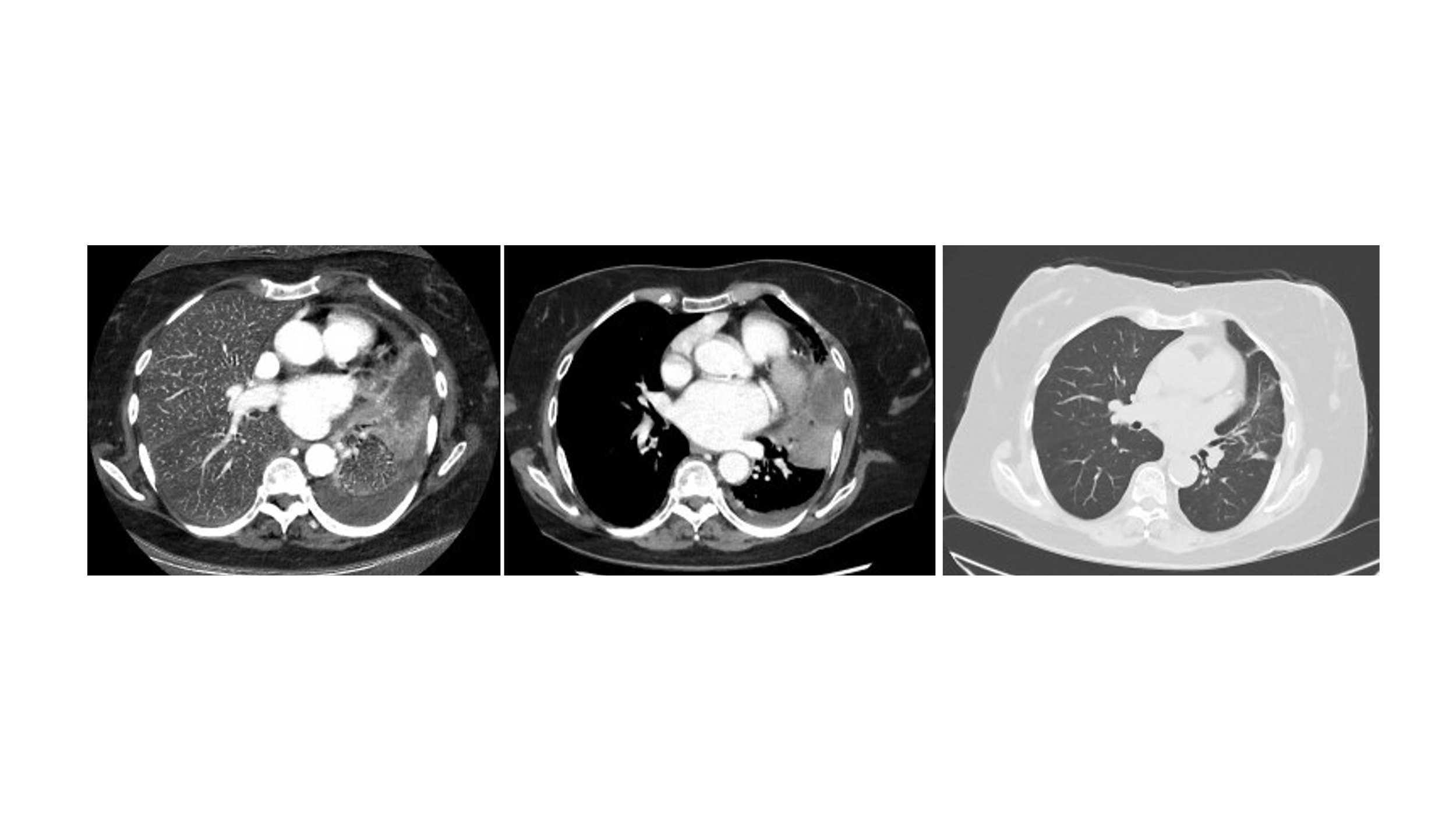


***Table legends***

***Supp. Materials Table 1 - Research strategy details***: The research strategy of each database and the interface or platform through which the database was searched is detailed in the table.

***Supp. Materials Table 2 - Selected articles***: Each included article is reported with the type of article and the number of included patients.

***Supp. Materials Table 3 - Antimicrobial regimens adaptation after diagnosis of Legionnaires’ Disease abscess***: the preferred monotherapy was a fluoroquinolone (11 patients treated); the preferred bi-therapy regimen was a combination of a fluoroquinolone and a macrolide (7 patients treated); three patients were treated with a triple therapy combining a fluoroquinolone, a macrolide, and rifampicin; AZM: azithromycin; CIP: ciprofloxacin; CLA: clarithromycin; LVX: levofloxacin; MFX: moxifloxacin; OFX: ofloxacin; PZFX: pazufloxacin; RIF: rifampicin; SPM: spiramycin.

***Supp. Materials Table 1***

| Database | Research strategy | Database accessed trough | Number of hits |
| --- | --- | --- | --- |
| Embase | (necrotizing pneumonia OR lung abscess* OR cavitary) AND (legionnaire* disease OR infection* legionella)  Selecting: “all fields”, “search as broad as possible” | Vrije Universiteit Brussel archive | 188 |
| Pubmed (Medline) | (necrotizing pneumonia OR lung abscess* OR cavitary) AND (legionnaire* disease OR infection* legionella)  Research in “advanced search”, selecting “all fields” | Vrije Universiteit Brussel archive | 94 |
| Web of Science | (necrotizing pneumonia OR lung abscess* OR cavitary) AND (legionnaire* disease OR infection* legionella)  Selecting: “all databases”,  Search on: “topic” | Vrije Universiteit Brussel archive | 135 |

***Supp. Materials Table 2***

| Year of publication | Study title | First author | Type of article | Number of patients described |
| --- | --- | --- | --- | --- |
| 2000 | Persistent Legionella infection in a patient after bone marrow transplantation | Schindel, C. et al. | Case report | 1 |
| 2001 | Legionella micdadei lung abscess in a patient with HIV-associated nephropathy. | Nzerue, C. Et al. | Case report | 1 |
| 2002 | Rapidly expanding lung abscess caused by Legionella pneumophila in immunocompromised patients: a report of two cases. | Miyara, T. et al. | Case series | 2 |
| 2004 | Cavitary Legionella pneumonia in a liver transplant recipient. | Fraser, T. G. et al. | Case report and narrative review | 1 |
| 2005 | Persistent culture-positive Legionella infection in an immunocompromised host | O'Reilly, S. et al. | Case report | 1 |
| 2006 | Cavitary Legionella pneumonia in a patient with immunodeficiency due to Hyper-IgE syndrome." | Di Stefano, F. et al. | Case report | 1 |
| 2007 | Community-acquired lung abscess caused by Legionella micdadei in a myeloma patient receiving thalidomide treatment. | Girard, L. P. et al. | Case report | 1 |
| 2007 | Legionella bozemanii, an elusive agent of fatal cavitary pneumonia. | Widmer, A. et al. | Case report | 1 |
| 2008 | Fatal coinfection with Legionella pneumophila serogroup 8 and Aspergillus fumigatus. | Guillouzouic, A. et al. | Case report | 1 |
| 2011 | Combined Legionella and Escherichia coli lung infection after a tsunami disaster. | Ebisawa, K. et al. | Case report | 1 |
| 2011 | Legionella pneumophila lung abscess associated with immune suppression. | Guy, S. D. et al. | Case report and narrative review | 1 |
| 2011 | Legionella jordanis in hematopoietic SCT patients radiographically mimicking invasive mold infection. | Meyer, R. et al. | Case series | 2 |
| 2013 | Legionellosis and lung abscesses: contribution of legionella quantitative real-time PCR to an adapted followup. | Descours, G. et al. | Case report | 1 |
| 2014 | A case of nosocomial Legionella pneumonia associated with a contaminated hospital cooling tower. | Osawa, K. et al. | Case report | 1 |
| 2015 | Legionella micdadei: A Forgotten Etiology of Growing Cavitary Nodules: A Case Report and Literature Review. | Lachant, D. et al. | Case report | 1 |
| 2016 | Persistant Legionella pneumophila and Enterococcus faecium pulmonary infection: Look for an abscess! | Gavand, P. E. et al. | Case report | 1 |
| 2018 | "Cavitary Pulmonary Nodules in an Immunocompromised Patient With Urothelial Carcinoma of the Bladder." | Morales, A. et al. | Case report | 1 |
| 2018 | Legionella indianapolisensis sp. nov., isolated from a patient with pulmonary abscess. | Relich, R. F. et al. | Case report | 1 |
| 2019 | "Pneumonia and pulmonary abscess due to Legionella micdadei in an immunocompromised patient." | Foissac, M. et al. | Case report | 1 |
| 2020 | Slowly or Nonresolving Legionnaires' Disease: Case Series and Literature Review. | Pouderoux, C. et al. | Case series and narrative review | 5 |
| 2021 | Legionella longbeachae pneumonia: Case report and review of reported cases in non-endemic countries. | Bell, H. et al. | Case report | 1 |
| 2021 | Legionella pneumophila as a cause of cavitary lung disease in systemic lupus erythematous. | Khokher, W. et al. | Case report | 1 |
| 2021 | Cavitary Legionella Pneumonia in AIDS: When Intracellular Immunity Failure Leads to Rapid Intrapulmonary Cavitation. | Durrance, R. J. et al. | Case report | 1 |

***Supp. Materials Table 2***

| Monotherapy | Bi-therapy | Triple-therapy |
| --- | --- | --- |
| LVX n:8 | LVX + SPM n:3 | RIF + ERY + LVX n:2 |
| AZM n:4 | RIF + CIP n:2 | RIF + SPM + LVX n:1 |
| MFX n:2 | LVX + AZM n:1 |  |
| CLA n:1 | CIP + AZM n:1 |  |
| PZFX n:1 | OFX + AZM n:1 |  |
|  | CIP + SPM n:1 |  |
|  | RIF + AZM n:1 |  |
|  | RIF + SPM n:1 |  |
